# MegaPath: sensitive and rapid pathogen detection using metagenomic NGS data

**DOI:** 10.1186/s12864-020-06875-6

**Published:** 2020-12-21

**Authors:** Chi-Ming Leung, Dinghua Li, Yan Xin, Wai-Chun Law, Yifan Zhang, Hing-Fung Ting, Ruibang Luo, Tak-Wah Lam

**Affiliations:** 1 Department of Computer Science, The University of Hong Kong, Pokfulam Road, Hong Kong, Hong Kong; 2 L3 Bioinformatics Limited, Rm 2114, Hong Kong Plaza, 188 Connaught Road West, Sai Ying Pun, Hong Kong

**Keywords:** Pathogen detection, Shotgun metagenomic sequencing, Next generation sequencing, Abundance detection, Read alignment

## Abstract

**Background:**

Next-generation sequencing (NGS) enables unbiased detection of pathogens by mapping the sequencing reads of a patient sample to the known reference sequence of bacteria and viruses. However, for a new pathogen without a reference sequence of a close relative, or with a high load of mutations compared to its predecessors, read mapping fails due to a low similarity between the pathogen and reference sequence, which in turn leads to insensitive and inaccurate pathogen detection outcomes.

**Results:**

We developed MegaPath, which runs fast and provides high sensitivity in detecting new pathogens. In MegaPath, we have implemented and tested a combination of polishing techniques to remove non-informative human reads and spurious alignments. MegaPath applies a global optimization to the read alignments and reassigns the reads incorrectly aligned to multiple species to a unique species. The reassignment not only significantly increased the number of reads aligned to distant pathogens, but also significantly reduced incorrect alignments. MegaPath implements an enhanced maximum-exact-match prefix seeding strategy and a SIMD-accelerated Smith-Waterman algorithm to run fast.

**Conclusions:**

In our benchmarks, MegaPath demonstrated superior sensitivity by detecting eight times more reads from a low-similarity pathogen than other tools. Meanwhile, MegaPath ran much faster than the other state-of-the-art alignment-based pathogen detection tools (and compariable with the less sensitivity profile-based pathogen detection tools). The running time of MegaPath is about 20 min on a typical 1 Gb dataset.

## Background

Detecting pathogens such as bacteria or viruses that cause infections such as pneumonia and meningitis is an important step in clinical diagnosis. One problem in detecting pathogens is that traditional methods of pathogen detection are time-consuming, as an infectious disease may be caused by a large range of pathogens, which have to be checked one by one. Another problem is that up to 60% of the pathogens in some infectious diseases cannot be detected [1]. This can cause a delay in treatment or even mistreatment of patients.

Unbiased next-generation sequencing (NGS) can detect DNA fragments (reads) of all species in a metagenomic sample with a mixture of different species. Those NGS reads can be classified into different taxa by comparing them with a collection of reference sequences, and pathogens can be detected if some reads match them. In clinical diagnoses, it is essential that a classifier can detect a significant number of reads supporting the potential pathogens and report as few false classifications as possible to give a high abundance rank to the pathogen. Otherwise, the pathogen cannot be distinguished from background noise, and it will take doctors a long time to go through a long list of candidates to dig out its existence.

Existing metagenomic classifiers are not effective for detecting low-similarity pathogens, i.e., pathogen with a genome that is not similar to the reference. This is because most classifiers detect pathogens by constructing a characteristic profile (e.g., *k*-mers) for each reference and assigning reads to species by comparing them with the reference profiles [2, 3]. When the characteristic profile does not match the genome of low-similarity pathogens, this approach fails and results in many incorrect or non-specific classifications.

Some tools assign reads to reference sequences by local or semi-global alignment. Using an alignment-based method, more reads can be assigned to the causal pathogen, but at the cost of much longer analysis time (over 4 h for a typical 1 Gb dataset). However, the alignment score of reads from a low-similarity pathogen is conceivably low, and these reads often cannot be assigned to the causal pathogen specifically, so the number of reads supporting the causal pathogen is still too low.

To detect low-similarity pathogens, we developed MegaPath for NGS-based pathogen detection. It has two significant contributions. First, instead of assigning each read to a reference sequence one by one, MegaPath analyzes all aligned reads globally to sort out a subset of reads with confident alignments. Then, MegaPath reassigns the non-specifically aligned reads to the species with confident alignments and discards spurious alignments to avoid potential false classifications. The reassignment increases the number of reads supporting the causal pathogens and reduces the number of false-positive assignments. Second, MegaPath implements a fast alignment-based approach, utilizing an enhanced maximum-exact-match prefix seeding strategy and a SIMD-accelerated Smith-Waterman algorithm.

Let us take a metagenomic NGS sample of cerebrospinal fluid [4] as an example. The similarity of the pathogen to reference is 18.9%. Centrifuge [2], CLARK [5] and Kraken [3], based on characteristic profile, detected 31, 1 and 6 reads from the pathogen, respectively. The abundance rank of the pathogen was 710, 1488 and 384, respectively. With that, a medical doctor needs to go through a list of hundreds of species to reach the causal pathogen. Kraken2 [3] is the successor of Kraken that applies more sophisticated characteristic profiles. It detected 74 reads from the pathogen and the abundance rank of the pathogen went up to 176. SURPI [6] spent four hours on read alignment and detected 76 reads from the pathogen, abundance rank at 264. In contrast, with better alignment tools and global analysis of reads, MegaPath took less than one hour and detected 608 reads for the pathogen, abundance rank at 33. In our experiment, MegaPath performed better than the existing tools, ran faster than the alignment-based tools and has comparable running time with the less sensitivity profile-based tools.

In addition to detecting pathogens with known reference sequences, MegaPath can detect novel pathogens without any similar DNA-level sequences in the reference database. MegaPath uses MegaHit [7] to assemble the reads from the novel pathogens to longer DNA fragments. Since protein sequences are better-conserved than DNA sequences [8, 9], these DNA contigs from novel pathogens are then annotated by DNA-protein alignment [10] to detect related species, genera or families.

### Implementation

**Fig. 1:**
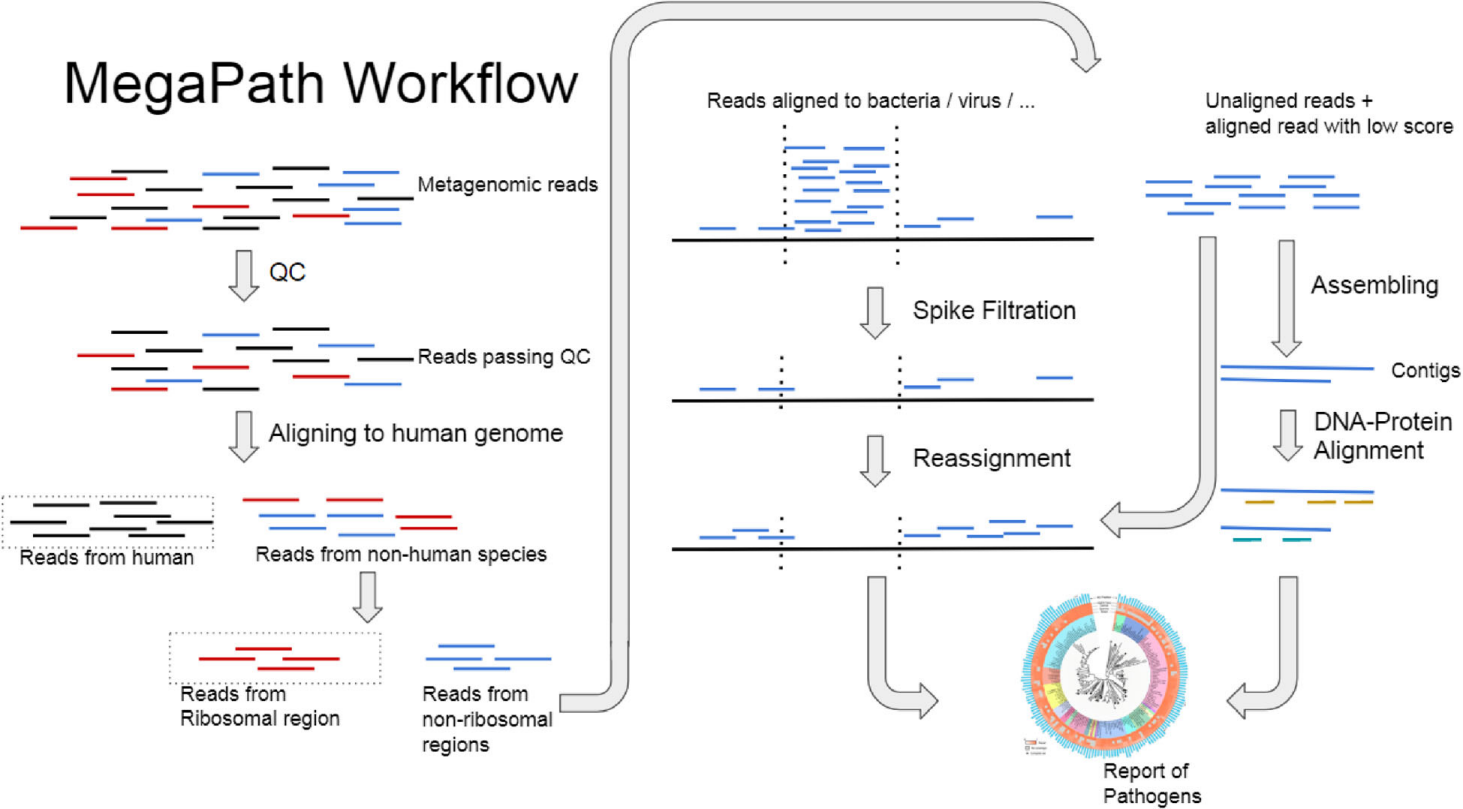
The workflow of MegaPath

Figure 1 shows the workflow of MegaPath. There are three major steps in MegaPath for detecting pathogens. First, it applies MegaGuide, an ultrafast NGS aligner specifically designed for pathogen detection to align reads to reference sequences of bacteria and viruses. Then it applies spike polishing to filter spurious alignments at highly repeated regions. Lastly, it applies a two-phase taxonomy assignment of reads.

#### Aligning reads with MegaGuide

MegaGuide is an ultra-fast aligner that follows a seed-and-extend procedure. In the seeding stage, MegaGuide searches for maximum-mappable seeds in the read using a BWT built from the reference sequences. The search will stop at (or a few bases after) a sequencing error or a genomic mutation. A new search will start at the end of the previous seed. The maximum-mappable seeding strategy reduces a vast number of short seeds that cannot be used to find the true alignments. The overlap between the seeds boosts sensitivity, especially for distant species. In the extension stage, MegaGuide implements an improved Smith-Waterman algorithm with each entry in the dynamic programming tables using 8 bits or less (instead of the normal implementation using 64 bits). Thus, by applying the 256-bit SIMD instructions, values of 32 entries can be calculated in parallel.

The reference database is large (the size of the latest RefSeq is over 30 Gb). It is neither efficient nor necessary to align all reads to all reference sequences. Thus, the following two types of reads, which are not informative for pathogen detection, will be filtered out from the downstream analysis. First, clinical metagenomic samples are usually dominated with human DNA (which could be as high as 95%) that carries no pathogen information. Second, short NGS reads sampled from the repetitive or homologous regions across species, e.g., ribosomal DNA, are not useful for pathogen detection. MegaPath filters out the two types of reads by aligning reads to the human reference genome and a database of homologous regions. Reads confidently aligned to the human reference genome or the homologous regions will be removed. The remaining reads will then be aligned to the pathogen genome database for pathogen detection.

#### Spike polishing

Aligning reads to human genome or homologous regions can detect most of the reads that are not informative for pathogen detection. However, not all of them can be detected due to the missing annotation or the incomplete classification of all possible homologous regions. To filter out reads sampled from unknown homologous regions, MegaPath makes use of the read depth information of each genome. Since MegaGuide aligns a read to all its possible genome positions, the read depth of the homologous regions is expected to be much higher than the other regions. MegaPath calculates the mean (*u*) and the standard deviation (*sd*) of the read depth of each genome. Continuous regions with a read depth higher than *u* + α·*sd* are defined as spike regions. All alignments in the spike regions will be removed from the downstream analysis. We have tried the value of α from 1 to 100. When α < 10, there are many regions misidentified as spike regions and are removed. When α > 50, only a few spike regions were detected and many homologous regions remain. The filtering has the best performance for α being from 10 to 50. Thus, we select α = 30 as default.

#### The two-stage taxonomy assignment algorithm

It is common that a read is aligned to different genomes with the same (or similar) confidence. These genomes may be from different species, genus, or even families. A straightforward way to assign a read to a taxon is assigning it to the lowest common ancestor (LCA) of all taxa the read aligned to. However, this approach leads to a large number of less specific assignments. MegaPath implements a two-stage assignment algorithm to increase the specificity. In the first stage, MegaPath assigns each read to the species they aligned to. We allow a read to be assigned to multiple species because the sequenced pathogen might have enriched mutations in some regions, and these regions can look very different from the correct reference genome. After assigning all the reads, in the second stage, MegaPath will try to reassign each of the shared reads to a species using the following rules.

First, all reads are assigned to one or more species according to their alignments. A read is tagged by ‘U’ if it is assigned to only one species, or ‘M’ if assigned to more than one species. Then, for each species, the numbers of ‘U’-tagged reads and the number of ‘M’-tagged reads are calculated. For a species *S*, we define UCount(*S*) as the number of ‘U’-tagged reads assigned to *S*, and AllCount(*S*) as the number of both ‘U’-tagged and ‘M’-tagged reads assigned to *S*. For two species *S* and *T*, we define MCount(*S, T*) as the number of reads assigned to both *S* and *T*. We say a species *S* weakly explains another species *T*, if 1) AllCount(*S*) - MCount(*S, T*) ≥ *r* * AllCount(*S*), and 2) UCount(*T*) < *e* * UCount(*S*). The default values of *r* and *e* are both 0.05. Descriptively, the criteria are interpreted as, 1) reads aligned to *S* are likely to be correct alignments (instead of misaligning read sampled from *T*) if quite a number of reads (*r* = 5%) are not similar to *T*; 2) the unique reads that support *T* might be a coincidence (*e* = 5%) due sequencing error or misalignment. We say *S* explains *T* if *S* weakly explains *T* and no species weakly explains *S*.

In the second stage, a read assigned to both *S* and *T* will be reassigned to *S* only if *S* explains *T*. After reassigning all the shared reads, MegaPath will apply the LCA algorithm to determine the taxon of each read.

## Results

Real datasets with known causal pathogens detected using traditional methods were used to evaluate the performance of MegaPath, and existing pathogen detection tools including SURPI [6], Centrifuge [2], CLARK [5], Kraken and Kraken2 [3]. Centrifuge, CLARK, Kraken, and Kraken2 construct a characteristic profile for each reference sequence and detect the existence of pathogens by comparing the reads to the constructed profiles. Kraken2 rans longer but performs better than its predecessor Kraken because it constructs a more sophisticated profile. These tools ran fast, but their sensitivity is not as good as the alignment-based tools’ for detecting low-similarity pathogens. SURPI detects pathogens by aligning reads to the reference sequences. SUPRI as the slowest tool, is generally more sensitive than the profile-based tools. MegaPath, by implementing a fast alignment strategy and analyzing the read alignments globally, achieves the highest sensitivity using a reasonable amount of running time.

We evaluated the tools using three types of datasets. First, we compared the sensitivity of the tools on real metagenomic datasets with known pathogens. Second, since the abundance rank of the pathogens in the real metagenomic datasets is unknown, we evaluated the tools based on mock metagenomic datasets with known relative abundance. Last, we evaluated the sensitivity and false-positive rate of the tools on detecting pathogens with different similarity to their corresponding reference sequence using a real cultured dataset.

### Performance on real metagenomic datasets

Nine real metagenomic datasets [4, 11, 12] were used to evaluate the sensitivity of MegaPath, SURPI [6], Centrifuge [2], CLARK [5], Kraken and Kraken2 [3] on detecting pathogens in real clinical samples. The datasets include cerebrospinal fluid, nasopharyngeal, and serum sample with the pathogen confirmed by conventional methods. Datasets 1 and 3 are two metagenomic NGS samples of cerebrospinal fluid (CSF) and nasopharyngeal (NP) swabs [4]. Datasets 2, 6, and 7 are plasma samples spiked with different concentrations of HIV [6]. Datasets 4, 5, 8, and 9 are HCV- or HBV-infected human livers [11].

Table 1 shows the number of reads and the abundance rank of the pathogen detected by each tool, sorted in increasing order of similarity between the pathogen genome and the reference sequence. Using BLASTn [13] as the aligner, the similarity is measured by the number of reads sampled from the pathogen that were aligned to the reference sequence against the number of reads sampled from the pathogen. Since the number of reads from the pathogen is unknown in the real dataset, we used those reads detected by the multiple tools as a rough estimation. Table 1 shows that when the pathogen genome is similar to the reference sequence (datasets 6 to 9), all tools performed quite well, except for dataset 7, in which the abundance of the pathogen is very low. For those datasets in which the pathogen genome is varied from the reference sequence (datasets 1 to 5), although most of the tools have detected more or less a few reads from the pathogen, the numbers were too low to tell apart the pathogen from the background noise, especially for those profile-based tools. Use dataset 1 as an example, Centrifuge, CLARK, and Kraken detected 31, 1, and 6 reads, respectively. The abundance rank of the pathogen was 710, 1488, and 384, respectively (a doctor will have to go through a list of over 300 candidate species to dig out the pathogen). Kraken2 outperformed its predecessor and detected 74 reads from the pathogen, with an abundance rank at 176. SURPI spent over four hours on read alignment and detected 76 reads for the pathogen, with an abundance rank at 264. Notably, MegaPath took less than one hour and detected 608 reads for the pathogen, with an abundance rank at 33. MegaPath performed the best among the existing tools but ran much faster than other alignment-based tools. Worth mentioning, the reference sequences of the low similarity Enterovirus D in dataset 1 and 3 were drafted and were just recently accepted to NCBI. We expect the performance of MegaPath as well as other tools will further improve using updated NCBI databases with more complete reference sequences.

**Table 1:** Benchmarking results of the pathogen detection tools on nine real metagenomic datasets

Dataset	Pathogens	Similarity to reference	MegaPath	SURPI	Centrifuge	CLARK	Kraken	Kraken2
			rank (# read)					
1	Enterovirus D	18.9%	**33 (608)**	264 (76)	710 (31)	1.5 K (1)	384 (6)	176 (74)
2	Human immunodeficiency virus	19.0%	**15 (3.8 K)**	63 (1.0 K)	78 (1.3 K)	69 (444)	109 (216)	32 (1.4 K)
3	Enterovirus D	27.9%	**622 (202)**	788 (50)	2.5 K (14)	2.6 K (8)	1.3 K (9)	1.5 K (17)
4	Hepatitis C virus	36.3%	**2 (12 K)**	2 (7.4 K)	65 (2.9 K)	15 (2.5 K)	3 (2.4 K)	31 (4.2 K)
5	Hepatitis C virus	38.4%	**2 (568)**	6 (374)	379 (150)	152 (182)	12 (130)	231 (218)
6	Human immunodeficiency virus	43.9%	**3 (54 K)**	5 (21 K)	6 (16 K)	4 (13 K)	6 (4.5 K)	2 (18 K)
7	Human immunodeficiency virus	46.1%	**187 (41)**	421 (19)	859 (18)	430 (18)	n/a (0)	496 (19)
8	Hepatitis B virus	69.4%	**2 (43 K)**	2 (33 K)	18 (19 K)	4 (20 K)	3 (19 K)	7 (20 K)
9	Hepatitis B virus	72.8%	**2 (3.0 K)**	4 (2.4 K)	191 (1.5 K)	41 (1.5 K)	4 (1.1 K)	94 (1.5 K)

### Performance on mock metagenomic datasets

The abundance rank of the pathogen is unknown in real metagenomic datasets. So in addition, we evaluated the performance of the tools using a mock metagenomic dataset, which was generated by mixing NGS reads from 3 real datasets: 1) 95% reads from a human sample (NA12878), 2) 4.75% (5% × 95%) reads from a metagenomic dataset with ten species with known abundance [14], and 3) 0.25% (5% × 5%) reads from a spiked bacteria. Since the reads from the species are known, the abundance rank of the spiked bacteria was determined as rank 4. Bacteria that have different similarities to their reference sequences were tested and the results are shown in Table 2.

**Table 2:** Benchmarking results of the pathogen detection tools on a mock metagenomic dataset

Dataset	Spiked bacteria	Similarity to reference	MegaPath	SURPI	Centrifuge	CLARK	Kraken	Kraken2
			rank (# read)					
1	Enterococcus hirae	20.6%	**13** (579)	96 (123)	18 **(7.3 K)**	386 (74)	65 (315)	83 (207)
2	Corynebacterium halotolerans	29.6%	**14 (526)**	n/a (0)	241 (331)	457 (58)	80 (205)	77 (220)
3	Clostridium botulinum	38.3%	**8 (1.7 K)**	62 (260)	65 (1.5 K)	108 (465)	65 (304)	37 (625)
4	Corynebacterium falsenii	42.1%	**9 (1.2 K)**	n/a (0)	208 (403)	n/a (0)	85 (192)	61 (328)
5	Gardnerella vaginalis	56.2%	**6 (20.2 K)**	23 (7.3 K)	14 (9.7 K)	17 (4.9 K)	13 (4.7 K)	14 (6.6 K)
6	*Pasteurella multocida*	60.4%	**6 (19.8 K)**	25 (2.9 K)	21 (6.2 K)	23 (2.8 K)	14 (3.6 K)	14 (5.6 K)
7	*Micrococcus luteus*	73.7%	**4 (25.5 K)**	14 (24.7 K)	10 (17.2 K)	13 (12.6 K)	11 (16.6 K)	11 (16.9 K)
8	Gallibacterium anatis	75.8%	**4 (37.4 K)**	11 (34 K)	10 (17.8 K)	12 (18.3 K)	11 (17.7 K)	11 (18 K)
9	Citrobacter freundii	77.6%	**4 (38.5 K)**	14 (21.4 K)	8 (27.2 K)	13 (10.4 K)	11 (18.6 K)	12 (18.6 K)
10	Haemophilus parainfluenzae	85.8%	**4 (38.7 K)**	n/a (0)	10 (18.0 K)	12 (20.2 K)	11 (17.4 K)	11 (18.8 K)
11	Leuconostoc gasicomitatum	91.6%	**4 (39.0 K)**	10 (38.4 K)	9 (21.8 K)	11 (22.6 K)	11 (21.4 K)	11 (21.5 K)
12	Human campylobacteriosis	95.6%	**4 (34.6 K)**	13 (28.1 K)	8 (30.6 K)	12 (19.0 K)	11 (19.2 K)	11 (18.4 K)

With high similarity between the bacteria and the reference sequence (datasets 5 to 12), most tools detected the spiked bacteria in the top 30 species. However, the abundance rank detected by other tools was incorrect. MegaPath detected the correct rank (rank 4 for datasets 7 to 12) or a close rank (rank 6 for datasets 5 and 6) for the spiked bacteria. A possible explanation is that other tools discard or randomly assign the reads without a unique alignment, which leads to error in the abundance rank. However, MegaPath analyzes all aligned reads globally and assigns the non-uniquely aligned reads based on reads that are confidently aligned, leading to a more accurate abundance rank. For low-similarity bacteria (datasets 1 to 4), the amount of non-unique alignments increased. Other tools failed or detected the bacteria with low abundance ranks. In contrast, MegaPath detected the spiked bacteria within the top 14 species.

We also evaluated the sensitivity, precision, and F1-score of the tools on the mock metagenomic dataset. The results are shown in Table 3. Since we know the origin of each read – from the human, the mock community or the spiked bacteria, a true positive is defined as a read being assigned correctly to its origin. Among the results, MegaPath achieved the highest sensitivity, precision, and F1 score. SURPI has been left out in Table 3, because it filtered out reads without annotation, making us unable to get the number of false-negative reads.

**Table 3:** Sensitivity, precision, and F1-score of the pathogen detection tools on a mock metagenomic dataset

Dataset	Spiked Bacteria	MegaPath	Centrifuge	CLARK	Kraken	Kraken2
		F1-score (Sensitivity, Precision)				
1	Enterococcus hirae	**98.5%** (97.1, 99.9%)	96.2% (92.8, 99.9%)	98.0% **(**96.1, 100%)	97.3% **(**94.8, 99.9%**)**	97.7% **(**95.6, 99.9%**)**
2	Corynebacterium halotolerans	**98.5%** (97.1, 100%)	96.3% **(**92.8, 100%**)**	98.0% (96.1, 100%**)**	97.3% **(**94.8, 100%**)**	97.8% **(**95.6, 100%**)**
3	Clostridium botulinum	**98.5%** (97.1, 100%)	96.3% **(**92.8, 99.9%**)**	98.0% **(**96.1, 100%**)**	97.3% **(**94.8, 100%**)**	97.8% **(**95.6, 99.9%**)**
4	Corynebacterium falsenii	**98.5%** (97.1, 100%)	96.3% **(**92.8, 100%**)**	98.0% **(**96.1, 100%**)**	97.3% **(**94.8, 100%**)**	97.8% **(**95.6, 100%**)**
5	Gardnerella vaginalis	**98.6%** (97.2, 100%)	96.3% **(**92.9, 100%**)**	98.0% **(**96.1, 100%,**)**	97.3% **(**94.8, 100%**)**	97.8% **(**95.7, 100%**)**
6	Pasteurella multocida	**98.6%** (97.2, 100%)	96.3% **(**92.9, 100%**)**	98.0% **(**96.1, 100%**)**	97.3% **(**94.8, 100%**)**	97.8% **(**95.7, 100%**)**
7	Micrococcus luteus	**98.6%** (97.2, 100%)	96.4% **(**93.0, 100%**)**	98.1% **(**96.2, 100%**)**	97.4% (94.9, 100%**)**	97.8% **(**95.8, 100%**)**
8	Gallibacterium anatis	**98.6%** (97.3, 100%)	96.4% **(**93.0, 100%**)**	98.1% **(**96.2, 100%**)**	97.4% **(**95.0, 100%**)**	97.8% **(**95.8, 100%**)**
9	Citrobacter freundii	**98.5%** (97.2, 99.9%)	96.4% **(**93.0, 99.9%**)**	98.0% **(**96.2, 99.9%**)**	97.4% **(**95.0, 99.9%**)**	97.8% **(**95.8, 99.9%**)**
10	Haemophilus parainfluenzae	**98.6%** (97.3, 100%)	96.4% **(**93.0, 99.9%**)**	98.1% **(**96.3, 100%**)**	97.4% **(**95.0, 100%**)**	97.8% **(**95.8, 100%**)**
11	Leuconostoc gasicomitatum	**98.2%** (96.6, 100%)	96.4% **(**93.0, 99.9%**)**	98.1% **(**96.3, 100%**)**	97.4% **(**95.0, 100%**)**	97.9% **(**95.8, 100%**)**
12	Human campylobacteriosis	**98.6%** (97.3, 100%)	96.4% (93.0, 100%**)**	98.1% **(**96.3, 100%**)**	97.4% **(**95.0, 100%**)**	97.8% **(**95.8, 100%**)**

### Sensitivity and FDR on mutated species

Since the real assignment to an exact species of an individual read is unknown in the real metagenomic datasets and the mock metagenomic dataset, thus it is unsuitable for evaluating the false discovery rate (FDR) of the tools. To evaluate the FDR of the tools, we have done an experiment on 139 NGS datasets of cultured isolated bacteria [2] where 1) all reads in each dataset are supposed to come from a single bacteria, and 2) the similarities are between 20 and 90%. According to the benchmarks in the previous two sections, we only benchmarked the two best performing tools Centrifuge and MegaPath in this section.

**Fig. 2:**
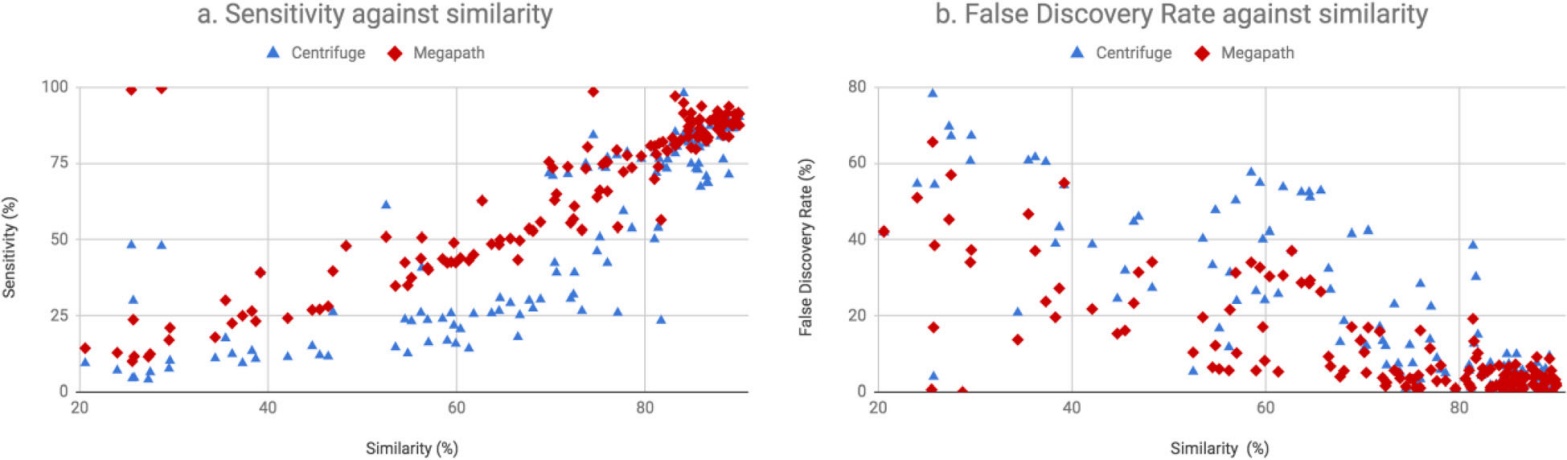
**(a)** Sensitivity and **(b)** False Discovery Rate (FDR) of Centrifuge and MegaPath

Figure 2 has shown the sensitivity (a) and FDR (b) of Centrifuge and MegaPath on each dataset for assigning reads to the known isolated bacteria. At the same similarity, higher sensitivity and lower FDR are expected. As shown, MegaPath consistently outperformed Centrifuge. Both the sensitivity and FDR of the two tools were close at higher similarities. However, at lower similarities, MegaPath achieved higher sensitivities and lower FDRs.

### Time consumption on real metagenomic datasets

The time complexities of the profile-based tools and the alignment-based tools are different. Profile-based tools including Centrifuge, CLARK, Kraken, and Kraken2, have a time complexity of *O*(*n + ts*), where *n* is the total number of bases of the input reads, *t* is the number of the input reads, and *s* is the number of species in the input database. Alignment-based tools including MegaPath and SURPI, have a time complexity of *O* (*nd*), where *d* is the maximum allowed edit distance between a read and a reference sequence.

To show the real-time consumption of each tool in practice, we benchmarked all tools using nine real metagenomic datasets. The results are shown in Table 4. All software tools expect for Kraken2 and CLARK were benchmarked using two Intel E5–2637 v2 (8 CPU cores) and 96 GB RAM. Kraken2 and CLARK asked for much memory, thus they were benchmarked on another faster machine with Intel E5–2695 v2 (24 CPU cores, but only 8 were used) and 192GB RAM. While the database indices could be reused once built, we did not include the time for building database indices in Table 4.

**Table 4:** Time consumption of six tools. SURPI was running in the “comprehensive mode”

Dataset	Pathogens	MegaPath	SURPI	Centrifuge	CLARK	Kraken	Kraken2
1	Enterovirus D	48 min	4 h 34 min	4 min	18 min	3 min	12 min
2	Human immunodeficiency virus	12 min	3 h 29 min	1 min	8 min	1 min	7 min
3	Enterovirus D	4 h 55 min	14 h 57 min	9 min	22 min	28 min	13 min
4	Hepatitis C virus	1 h 45 min	5 h 13 min	3 min	10 min	12 min	6 min
5	Hepatitis C virus	1 h 18 min	4 h 6 min	3 min	14 min	10 min	9 min
6	Human immunodeficiency virus	26 min	3 h 51 min	1 min	7 min	1 min	12 min
7	Human immunodeficiency virus	11 min	3 h 22 min	1 min	8 min	1 min	15 min
8	Hepatitis B virus	58 min	5 h 9 min	7 min	12 min	9 min	28 min
9	Hepatitis B virus	1 h 4 min	4 h 20 min	4 min	13 min	9 min	1 h 11 min

## Discussion

Next-generation sequencing (NGS) has enabled unbiased detection of pathogens through mapping the sequencing reads of a patient sample to the known reference sequence of bacteria and viruses. However, existing NGS-based pathogenic detection tools fail to detect low-similarity pathogens or usually assign them with a low rank because the tools fail to assign reads to the reference sequences correctly. In this paper, we introduced MegaPath for detecting these low-similarity pathogens. MegaPath analyzes read alignments globally and uses a two-phase assignment of reads. We will discuss the performance of the two-phase assignment design in this section. Besides, for those novel pathogens without a reference sequence, we will discuss how MegaPath can detect them by sequence assembling and how good its performance is.

### Performance of the two-phase assignment of reads

As the mutation rate of bacteria and viruses is high, and the sequence of some pathogens are unknown, many pathogens are without a similar reference sequence in the database. Existing pathogen detection tools often discard a read sampled from low-similarity regions or assign the read to an arbitrary position. As a result, a low-similarity pathogen may not be detected or may be detected at a low abundance rank. To solve the problem, MegaPath applies a two-phase assignment of reads. To evaluate the performance of the two-phase assignment design, we ran MegaPath with and without a two-phase assignment on nine real metagenomic datasets.

The results are shown in Table 5. In datasets 1, 3, and 6, the number of reads assigned to the pathogen increases with the two-phase assignment because several non-uniquely aligned reads from low-similarity regions have been reassigned to the correct pathogen. Two-phase assignment not only increases the number of reads assigned to the correct pathogen, but also reduces false-positive alignments. In datasets 2, 4 and 7, although the number of reads assigned to the correct pathogen did not change, the number of false-positive reads aligned to species with a higher abundance rank is reduced. As a result, the abundance rank of the correct pathogen increases. For pathogens similar to the reference sequence (datasets 8 and 9), the effect of the two-phase assignment is insignificant because most of the reads sampled from the pathogen were aligned correctly.

**Table 5:** Benchmarking results of MegaPath with and without a two-phase assignment on nine real metagenomic datasets

Datasets	Pathogens	Similarity to reference	MegaPath (w/ two-phase assignment)	MegaPath (w/o two-phase assignment)
			rank (# read)	
1	Enterovirus D	18.9%	**33 (608)**	50 (594)
2	Human immunodeficiency virus	19.0%	**15** (3.8 K)	23 (3.8 K)
3	Enterovirus D	27.9%	**622 (202)**	631 (197)
4	Hepatitis C virus	36.3%	**2** (12 K)	4 (12 K)
5	Hepatitis C virus	38.4%	2 (568)	2 (568)
6	Human immunodeficiency virus	43.9%	3 **(54.2 K)**	3 (54.0 K)
7	Human immunodeficiency virus	46.1%	**187** (41)	347 (41)
8	Hepatitis B virus	69.4%	2 (43 K)	2 (43 K)
9	Hepatitis B virus	72.8%	2 (3.0 K)	2 (3.0 K)

### Detecting novel species without a reference

When a pathogen is novel (i.e., without a reference sequence in the database), detecting the pathogen using alignment does not work. MegaPath handles such cases by performing sequence assembly using MegaHit [9]. MegaPath assembles the unaligned reads and the reads aligned to viruses, which are known to mutate rapidly, to construct contigs for novel species.

We evaluated the performance of MegaPath in detecting a novel pathogen using a serum sample from a patient infected with a novel BASV Rhabdovirus [15]. To simulate a situation in which BASV is a novel virus, the DNA and protein sequences of BASV, which were in the reference sequence database, were removed. As a result, eight contigs were assembled by MegaPath. The longest contig was 3131 bp, with a total length of 11,155 bp, which is close to the average genome size of a virus [16]. SURPI also supports sequence assembly for discovering novel species. It assembled 15 contigs, the longest contig was 1726 bp, with a total length of 11,019 bp, both are shorter than MegaPath. All these contigs are correct because they can be aligned to the reference sequence of the BASV Rhabdovirus.

## Conclusions

Next-generation sequencing (NGS) enables unbiased detection of pathogens that cannot be achieved using traditional methods, including culture and PCR. Typical NGS approaches for detecting a pathogen require mapping the sequencing reads of a patient sample to the pathogen’s reference sequences. However, when the target pathogen has no close relative with a known reference sequence, or when the pathogen has a high load of mutations compared to its predecessor, read mapping often fails due to low similarity between the pathogen and the reference sequence. As a result, the detection of such pathogens remains ineffective.

To solve the problem, we developed MegaPath that performs global analysis of aligned reads in order to increase the mapping rate and mapping quality of reads to reference sequences, especially for low-similarity pathogens. This process is computationally intensive, but thanks to MegaPath’s highly optimized alignment engine (MegaGuide), fast and accurate detection of pathogens was made possible. MegaPath can detect low-similarity pathogens in a typical metagenomic dataset in around 20 min.

### Availability and requirements

Project name: Megapath Project

Project home page: https://sourceforge.net/projects/megapath

Operating system(s): Linux

Programming languages: Perl, BASH, C/C++

Other requirements: docker engine v17.12.0-ce, AVX2 (CPU)

License: GNU GPLv3

Any restrictions to use by non-academics: Nil

## Data Availability

The datasets generated and/or analysed during the current study are available at https://sourceforge.net/projects/megapath.

## Abbreviations

BASV: BAS-congo Virus
CSF: Cerebrospinal Fluid
DNA: DeoxyriboNucleic Acid
HBV: Hepatitis B Virus
HCV: Hepatitis C Virus
HIV: Human Immunodeficiency Virus
FDR: False Discovery Rate
LCA: Lowest Common Ancestor
NGS: Next Generation Sequencing
NP: nasopharyngeal
SURPI: Sequence-based UltraRapid Pathogen Identification
